# Oculomotor dynamics in emmetropes, myopes, and hyperopes: A behavioral perspective

**DOI:** 10.1371/journal.pone.0339294

**Published:** 2025-12-22

**Authors:** Suraj Upadhyaya

**Affiliations:** Chicago College of Optometry, Midwestern University, Downers Grove, Illinois, United States of America; L V Prasad Eye Institute, INDIA

## Abstract

**Purpose:**

The oculomotor system, which controls eye movements, is closely linked to visual processing. While refractive errors are common, their influence on oculomotor behavior remains underexplored. This study compared oculomotor performance among emmetropic, myopic, and hyperopic individuals.

**Methods:**

In this cross-sectional, single-visit study, 67 participants (33 myopes, 10 hyperopes, 24 emmetropes; mean age 25.9 ± 3.0 years) completed fixation and visually guided saccade tasks at a viewing distance of 57 cm. A centrally positioned black, disc-shaped target (0.50° in diameter) was displayed on the screen for 45 seconds, after which it shifted to a predetermined location to elicit visually guided saccades. Clinical measures were included in the correlation analysis to ensure the findings were clinically relevant and to examine relationships between research variables and patient outcomes. Eye movements were recorded using the EyeLink 1000Plus. Fixation stability was quantified using Bivariate Contour Ellipse Area (BCEA). Fixational saccade metrics, vergence stability, and saccadic behavior were analyzed. Axial length and corneal power were measured using a portable ultrasound scanner.

**Results:**

Fixation stability differed significantly across groups, with myopes exhibiting larger BCEA values compared to emmetropes (H[2] = 10.6, p < 0.05). Analysis of fixational saccades revealed that myopes demonstrated significantly greater amplitude (H[2] = 507.4, p < 0.001), peak velocity (H[2] = 595.7, p < 0.001), and frequency (H[2] = 9.0, p < 0.05) relative to the other groups. Vergence stability was also poorer in myopes than in emmetropes (H[2] = 8.7, p < 0.05). In contrast, saccadic behavior during visually guided tasks showed no significant group differences in latency (H[2] = 1.9, p = 0.38) or gain (H[2] = 5.59, p = 0.06). Finally, correlation analyses indicated no significant associations between fixation stability and spherical equivalent, axial length, or corneal power.

**Conclusion:**

Myopes exhibited more unstable fixation, characterized by larger, faster, and more frequent fixational saccades. These findings suggest distinct oculomotor behavior in myopia, potentially reflecting altered spatiotemporal processing during visual development.

## Introduction

Efficient oculomotor control is vital for maintaining stable and accurate visual perception during a wide range of visual tasks. This control system relies on the precise coordination of subtle eye movements—such as saccades, fixations, and vergence—regulated by the central nervous system to ensure visual stability and performance. Visual fixation is essential for maintaining a steady gaze on a target, allowing the eyes to continuously sample fine visual details at the fovea. This process supports high-acuity vision and prevents visual fading [1–7].

Fixation stability is a complex interplay of microsaccades, drift, and tremor—collectively known as fixational eye movements [8,9]. Microsaccades actively contribute to visual perception by precisely stabilizing gaze within the fovea, aligning with shifts in spatial attention, and facilitating the exploration of fine spatial detail in complex scenes under natural viewing conditions [10–12]. Disruptions in fixation stability have been linked to diminished visual acuity and impaired performance in tasks requiring sustained visual attention [13–16]. Fixation has been shown to be impaired in amblyopes, strabismus, cerebral impairments, and many other neurodevelopmental disorders [17–24].

Visually guided saccades are large, goal-directed eye movements that follow a predictable relationship between amplitude, velocity, and duration, known as the main sequence. Monitoring deviations from this pattern is important because such irregularities can serve as indicators of neurological or visual dysfunction, making their assessment valuable for both clinical diagnostics and research [25,26]. Emerging evidence suggests that refractive errors can influence oculomotor behavior, particularly in areas such as fixation stability, vergence control, and microsaccadic dynamics. Refractive errors can influence several aspects of visual function by reducing retinal image quality and disturbing binocular alignment. Optical blur and higher-order aberrations compromise image clarity, which affects the sensory input needed for accurate eye movement control [27]. Conditions such as anisometropia can alter the accommodation–vergence relationship, creating binocular vision stress and reducing stereoacuity [28,29]. These changes may lead to reduced fixation and vergence stability, and while direct evidence for saccadic changes is limited, the impact of binocular stress suggests possible effects on saccadic accuracy and timing [30,31]. Although prior studies have examined fixation instability in myopes, a significant gap persists in the literature. A comprehensive, comparative analysis across all major refractive groups—myopes, hyperopes, and emmetropes—within a single, controlled framework is lacking. For example, Ghasia et al. studied fixational eye movements in 10 myopes over a decade ago, but without a refractive error-based comparison group, limiting the comparability of their findings [32]. Shaikh et al. included a control group, yet only one true myope was represented, and the presence of amblyopia—a known confounder of fixational saccades—further compromised interpretability [22]. Tang et al. investigated 14 myopes and found no significant differences in saccadic behavior between corrected and uncorrected conditions, but their study excluded hyperopes and emmetropes and did not assess vergence stability [33]. While Tsang and Ghasia have contributed valuable data on oculomotor behavior, neither addressed fixation stability directly. Coletta’s abstract hinted at potential differences in fixation stability, but was based on a small sample of ten subjects with undisclosed refractive status, limiting both transparency and generalizability [34]. Moreover, these findings have not been independently validated in peer-reviewed literature. Crucially, none of these studies simultaneously compared all three refractive groups while incorporating vergence stability—a key aspect of binocular coordination and visual function. Although fixation control mechanisms have been well-characterized in individuals with normal vision, less is known about how these mechanisms differ across refractive error groups—namely, myopes, hyperopes, and emmetropes [34–38]. Refractive errors, which result from the eye’s inability to focus light accurately on the retina, are becoming increasingly prevalent worldwide. Myopia is typically associated with an elongated axial length and blurred distance vision, while hyperopia involves a shorter axial length and increased accommodative demand for near tasks [39,40]. Emmetropes, by contrast, maintain a balanced relationship between axial length and refractive power.

Given the growing global burden of refractive errors—particularly myopia—it is essential to understand their broader impact on visual function. While the neurophysiology of eye movements has been widely studied, relatively few investigations have systematically examined how refractive status affects specific oculomotor metrics such as saccadic latency, fixation dispersion, vergence stability, and fixational saccades.

This study addresses this gap by systematically comparing key oculomotor parameters—saccadic latency, saccadic gain, fixation stability, vergence stability, and fixational saccades activity—across individuals with myopia, hyperopia, and emmetropia in one setting. By including hyperopes, who are often underrepresented in vision science research, and emphasizing vergence stability, this study offers a more comprehensive understanding of how refractive errors shape oculomotor function. Identifying these differences may enhance clinical assessments, improve visual training protocols, and deepen our understanding of oculomotor adaptations in ametropic individuals.

## Methods

### Participants and experimental paradigms

This cross-sectional prospective study was approved by the Institutional Review Board of Midwestern University (IRB no: 23042) and conducted at the Chicago College of Optometry, Downers Grove, Illinois, USA, between Oct 2023 and January 2025. Informed written consent was obtained from all participants after a detailed explanation of the procedures, and the study adhered to the principles of the Declaration of Helsinki.

Participants included individuals both with and without refractive errors, provided their astigmatism was less than 0.50 diopters (D). All participants demonstrated 20/20 visual acuity—either corrected or uncorrected—as verified using a standard Snellen chart. To minimize confounding effects on oculomotor behavior, individuals with pathological myopia (defined as high myopia accompanied by structural retinal changes) were excluded. Additional exclusion criteria included any history of neurological disorders, ocular diseases (such as strabismus, amblyopia, anisometropia or glaucoma), prior ocular surgery, or use of medications known to affect the central nervous system. During testing, all participants wore soft contact lenses to ensure consistency in visual correction.

Eye movements were recorded using the EyeLink 1000plus (SR Research, Ontario, Canada), a non-invasive 1000 Hz infrared tracker with 0.01° spatial resolution, enabling accurate detection of saccades as small as 0.10°. Both the pupil center and corneal light reflex were used as references, a method known as the Pupil-Corneal Reflection (P-CR) technique in EyeLink. By tracking the relationship between the two—specifically, the vector from the corneal reflection to the pupil center—the system can accurately calculate gaze direction, as the pupil moves relative to the fixed corneal reflection. A chin and forehead rest was used to minimize head movement, and an additional head strap was employed to stabilize the head position further, thereby enhancing the precision of small eye movement recordings. Fixation targets were displayed on a 2048 × 1152 pixels display with 60 Hz refresh rate at 57 cm viewing distance. A nine-point monocular calibration and validation procedure was ensured before every experiment.

Participants binocularly fixated on a centrally located black disc-shaped target (30 arcminutes in diameter) displayed on the screen. Each participant completed 12 trials, each lasting 45 seconds. The first 35 seconds of each trial were used for fixation analysis, following a 10-second interval separating fixation and saccade data. After the 45-second fixation period, the central target disappeared and reappeared at one of several predetermined eccentric locations—horizontally and vertically—at amplitudes of ±5°, ± 10°, or ±15°. Eccentric targets were presented in a pseudorandomized and counterbalanced sequence to prevent predictability and minimize anticipatory responses. Each eccentric target remained visible for 500 milliseconds before end of the trial. This target shift elicited a visually guided saccade, enabling assessment of saccadic performance. Visually-guided saccades were defined as eye movements directed toward peripheral targets with a minimum amplitude of 3.5°, reflecting the spatial distance between the central fixation point and the peripheral stimulus. Saccades were excluded if they were anticipatory (<100 ms) [41–43], in the incorrect direction, or occurred during blinks or tracking loss. Corneal power (K1: steepest meridian power, K2: flattest meridian power) was measured using the PalmScan K2000 and Micro Medical Devices (MMD) keratometer, which averaged five readings per eye. Axial length was measured with the A2000 A-scan mode (MMD), averaging 10 measurements per eye. The spherical equivalent (SE) was calculated as the spherical power plus half of the cylinder power. Hyperopia was defined as SE ≥ +0.50 D, emmetropia as SE between +0.49 D and −0.49 D, and myopia as SE ≤ −0.50 D, consistent with American Academy of Ophthalmology guidelines [44].

### Data acquisition and analysis

Raw eye movement data from the EyeLink 1000plus were analyzed using a custom MATLAB script (MathWorks, Natick,MA,USA). Visually guided saccades were identified by differentiating position data into velocity, applying a 50 deg/sec threshold for saccade onset and offset. Saccade latency was calculated as the time interval (in milliseconds) between the onset of the peripheral visual target and the onset of the visually guided saccade. Blink correction was applied on raw data which removed 100 msec of data before and after each blink to account for missing pupil information and avoid spurious position changes. Evaluating visually guided saccades prior to fixation analysis ensures baseline oculomotor integrity across refractive groups. Saccades reflect core neuromuscular and neural functions essential for visual exploration and attentional control. Confirming that saccadic dynamics follow expected patterns—such as the main sequence—helps validate that any observed fixation differences are not confounded by underlying motor deficits. This step strengthens the reliability of subsequent micro-level eye movement analyses. Fixational saccades were detected using a velocity-based method: movements exceeding 5 × baseline velocity SD, lasting over 6 ms, and occurring simultaneously in both eyes are flagged as actual events using the Engbert and Kliegl algorithm [8,9,45–47]. Metrics analyzed included radial amplitude (°), peak velocity (°/s), and fixational saccade rate (Hz). Radial fixational saccades amplitude refers to the distance between the start and end points of a fixational saccade, representing its magnitude in visual space. Peak fixational saccade velocity is the highest speed reached during a fixational saccade, typically measured in degrees per second (°/s). Fixational saccade rate indicates how frequently these fixational saccades occur, expressed as fixational saccades per second (Hz). Events smaller than 0.10° were excluded as noise. Fixation stability was assessed via the BCEA, which captures 68.2% of the horizontal and vertical dispersion of eye movement during central fixation [48,49]. BCEA was calculated as:


$$\text{BCEA}=\text{2}.\text{291}\times \pi \times \sigma \text{x}\times \sigma \text{y}\times \sqrt{\left(I-\rho^{\text{2}}\right)}$$

In this equation, σx and σy represent the standard deviations of horizontal and vertical eye positions from the central fixation point, respectively, while p denotes the Pearson correlation coefficient between the two axes. The constant 2.291 corresponds to the chi-square value for a 68.2% probability contour, providing a standardized measure of fixation dispersion. Lower BCEA values reflect greater stability [14,50,51]. BCEA has been widely applied in vision research [14,18–21,51–53]. We randomly selected right-eye position data for fixation and saccade analyses. Vergence stability was calculated as the difference between the left and right eye gaze positions. Specifically, we measured horizontal and vertical gaze disparities between the left and right eyes over the fixation period to assess binocular alignment consistency. This vergence stability metric was derived from binocular data using the BCEA formula. Saccadic gain was calculated as the ratio of actual saccade amplitude to target amplitude. Statistical analyses were performed using Kruskal-Wallis one-way ANOVA on ranks, followed by Dunn’s post hoc tests with correction for multiple comparisons. Spearman’s rank correlation assessed associations between fixation stability and clinical or oculomotor measures. All statistical tests were performed in SigmaPlot 16.0, with a significance threshold set at α = 0.05.

## Results

### Participant demographics and clinical profiles

A total of 67 participants were included in the study: 33 myopes, 10 hyperopes, and 24 emmetropes. The mean age was 26.30 ± 3.66 years for myopes (range: 20–38), 26.90 ± 2.18 years for hyperopes (range: 24–31), and 25.00 ± 2.07 years for emmetropes (range: 22–32). A Kruskal-Wallis one-way ANOVA on ranks revealed no statistically significant difference in age among the groups (H[2] = 5.56, p = 0.06). Gender distribution was as follows: 69.7% female and 30.3% male in the myopic group; 60% female and 40% male in the hyperopic group; and 54.2% female and 45.8% male in the emmetropic group. The ethnic composition of the total sample was 35.8% Asian, 2.9% Black, 4.4% Hispanic, and 56.6% White. Refractive and biometric data showed that myopes had a mean spherical equivalent (SE) of −3.11 ± 1.92 D (range: −10.25 to −0.75 D), axial length of 24.67 ± 0.80 mm (range: 23.29–26.48 mm), and keratometry readings of 43.44 ± 1.87 D (K1) and 42.99 ± 1.67 D (K2). Hyperopes had a mean SE of +0.90 ± 0.78 D (range: + 0.50 to +3.00 D), axial length of 23.08 ± 0.82 mm (range: 21.88–24.30 mm), and keratometry readings of 42.61 ± 1.27 D (K1) and 42.49 ± 1.17 D (K2). Emmetropes had a mean SE of +0.07 ± 0.13 D (range: −0.25 to +0.25 D), axial length of 23.84 ± 0.52 mm (range: 22.86–25.13 mm), and keratometry readings of 42.94 ± 1.31 D (K1) and 42.49 ± 1.60 D (K2). All participants had corrected visual acuity of 20/20.

**Fig 1:**
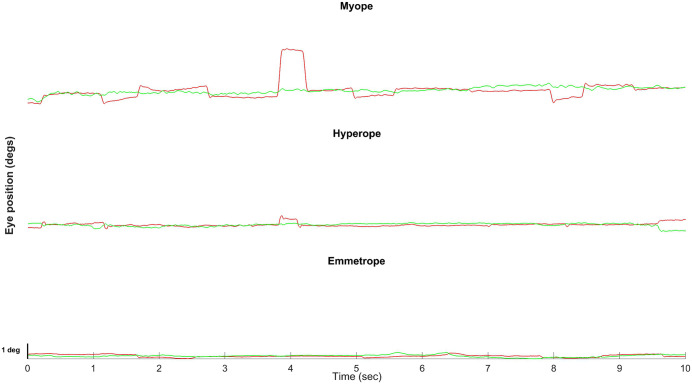
Raw data showing horizontal (red) and vertical (green) eye positions of representative myopic, hyperopic, and emmetropic participants (based on spherical equivalent) while fixating a 0.5° disk-shaped target. The x-axis represents time in seconds, and the y-axis represents eye position in degrees.

Fig 1 displays representative horizontal (red) and vertical (green) raw eye position data over a 10-second trial for one subject from each refractive group while fixating on a 0.5° black disk-shaped target on a white background. The myopic subject had a spherical equivalent refractive error of −1.75 D. The hyperopic subject had a refractive error of +1.00 D, and the emmetropic subject had a refractive error of 0.00 D.

### Visually guided saccades

**Fig 2:**
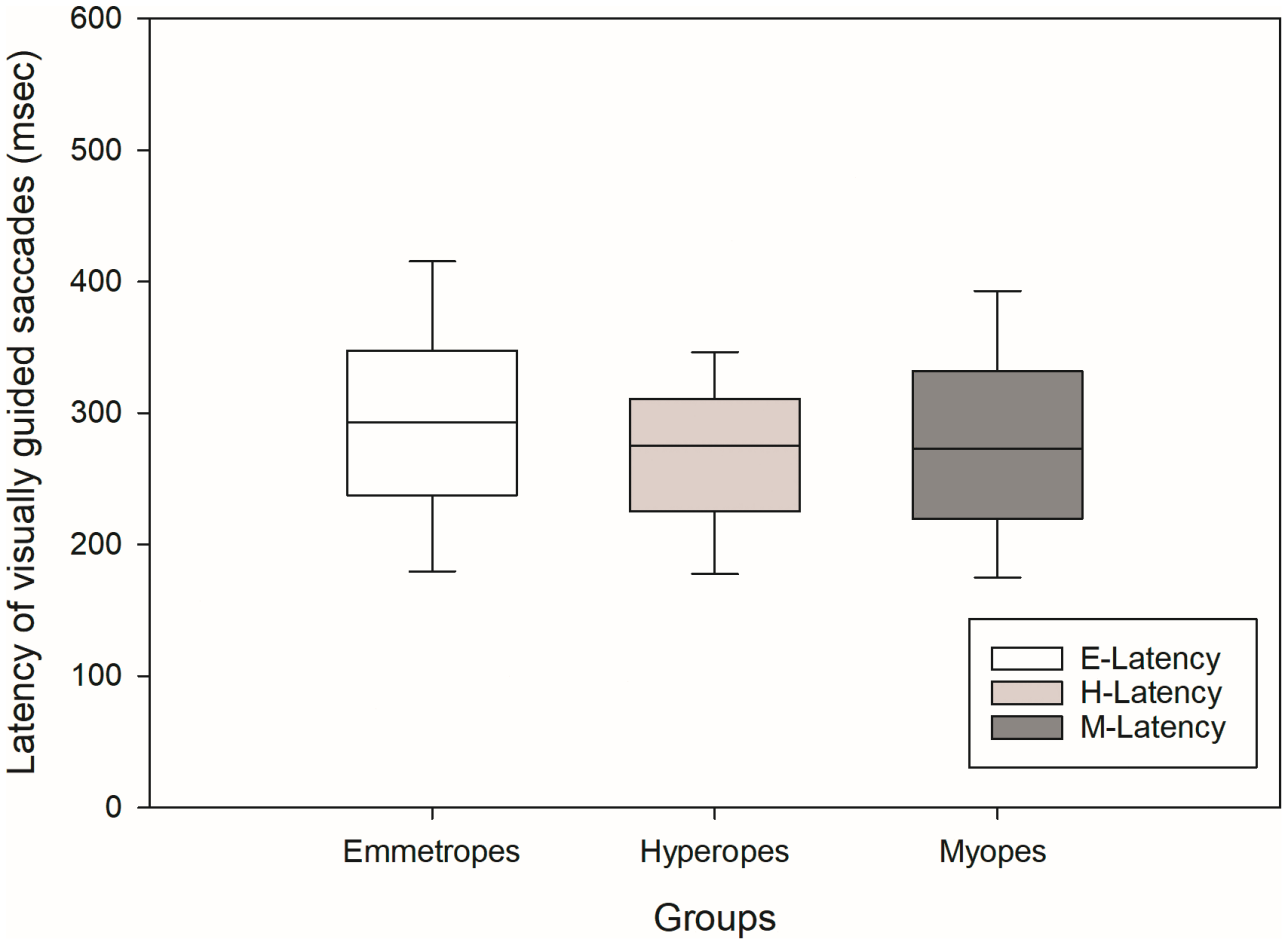
Box plot showing the distribution of latency of visually guided saccades (ms) for myopes, hyperopes, and emmetropes. The edges of the box represent the 25th and 75th percentiles; the solid black line indicates the median. Whiskers extend to the most extreme non-outlier values.

**Fig 3:**
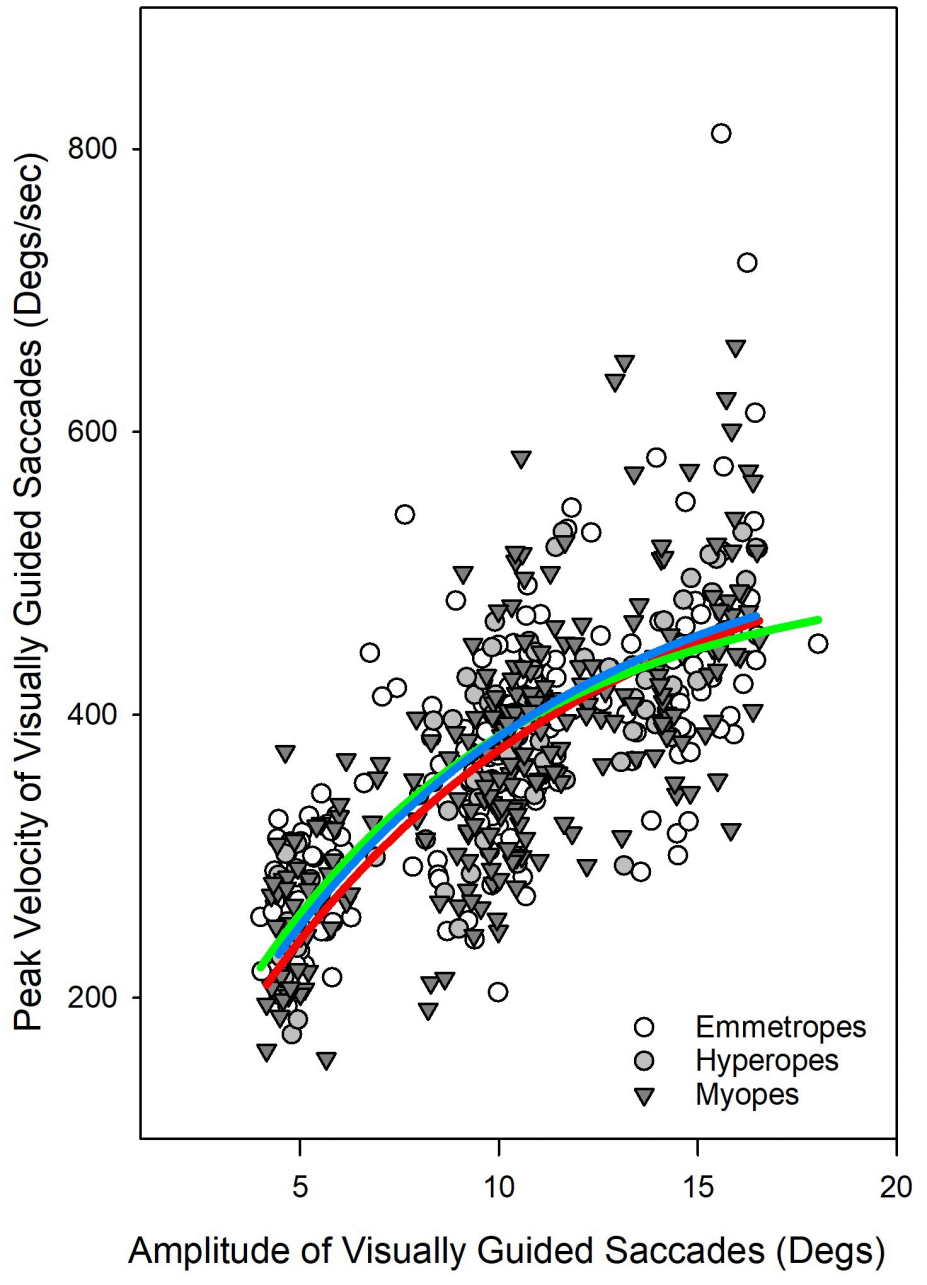
Scatter plot depicting the relationship between amplitude (°) and peak velocity (°/s) of visually guided saccades for myopes, hyperopes, and emmetropes. An exponential rise-to-maximum function was fitted to the data for each group. The fitted slopes are: Myopes (0.14), Hyperopes (0.12), and Emmetropes (0.13). The red line represents the myopic group, the blue line represents the hyperopic group, and the green line represents the emmetropic group.

**Fig 4:**
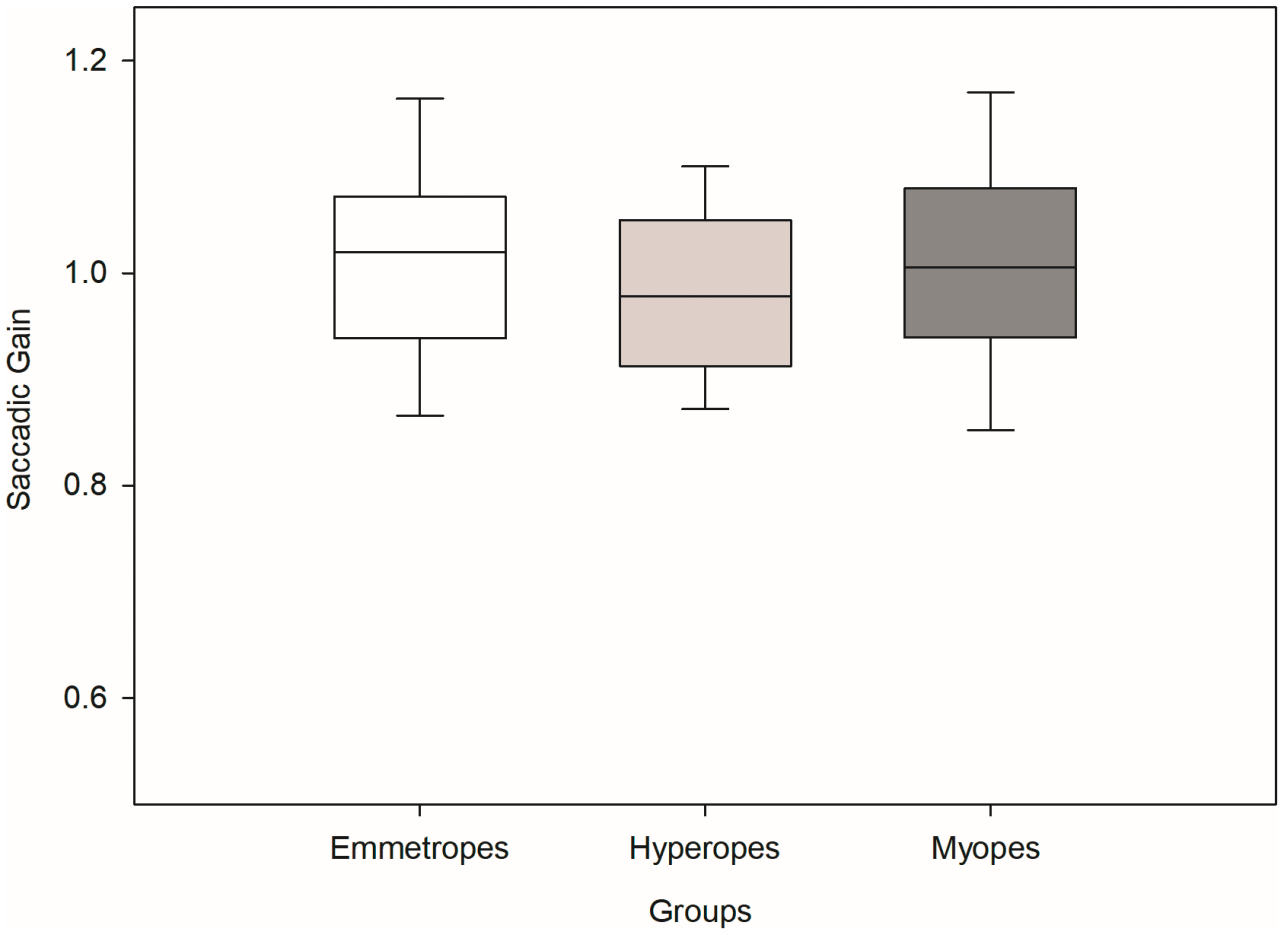
Box plot showing the distribution of saccadic gain for visually guided saccades in myopes, hyperopes, and emmetropes. The edges of the box represent the 25th and 75th percentiles; the median is shown by a solid black line. Whiskers extend to the most extreme non-outlier values.

First, we looked at the saccadic latency in these three groups. Saccadic latency (Fig 2) showed median values(Intra quartile range (IQR)) of 273(112.5) ms for myopes, 275(86) ms for hyperopes, and 293(110) ms for emmetropes. A Kruskal-Wallis ANOVA on ranks indicated no significant group differences (H[2] = 1.9, p = 0.38). We also looked at the relationship between amplitude and peak velocity of visually guided saccades among these groups. Main sequence analysis (Fig 3) revealed that all groups followed the expected relationship between saccade amplitude and peak velocity. No significant differences were found in saccadic amplitude Kruskal-Wallis ANOVA on ranks (H[2] = 0.15, p = 0.90) or peak velocity (H[2] = 1.17, p = 0.50). And finally, we looked at saccadic gain of visually guided saccades in these three groups. Saccadic gain (Fig 4) was also comparable across groups. The median gain was 1.02(0.13) for myopes, 0.98(0.14) for hyperopes, and 1.00(0.14) for emmetropes. Although the myopic group showed slightly higher gain, the difference was not statistically significant Kruskal-Wallis ANOVA on ranks (H[2] = 5.59, p = 0.06).

### Fixation and vergence stability

**Fig 5:**
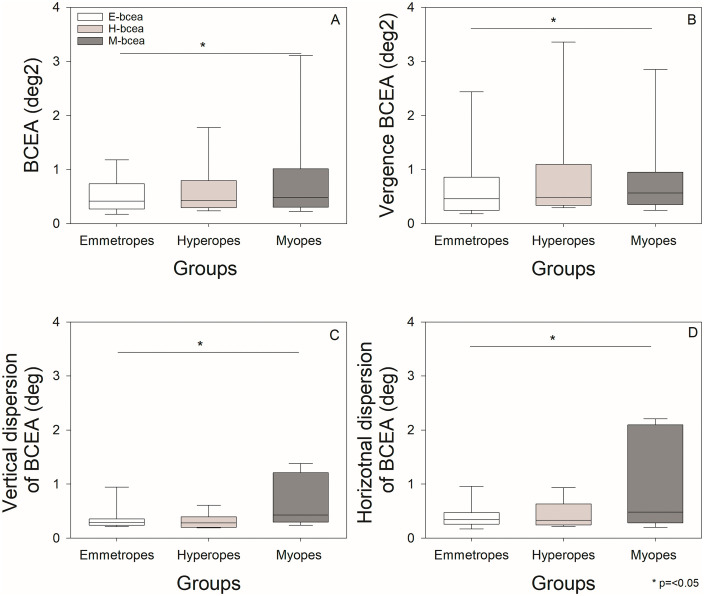
Box plots illustrating fixation and vergence stability metrics for myopes, hyperopes, and emmetropes: (A) BCEA (deg²), (B) V-BCEA (deg²), (C) Vertical dispersion (deg), and (D) Horizontal dispersion (deg). The edges of the box represent the 25th and 75th percentiles; medians are shown by solid black lines. Whiskers extend to the most extreme non-outlier values. Asterisks (*) indicate statistically significant differences.

Table 1 and Fig 5 summarize the fixation and vergence stability matrix seen in this cohort. Fixation stability, measured by BCEA, differed significantly among groups Kruskal-Wallis ANOVA on ranks (H[2] = 10.61, p = 0.005). Post hoc Dunn’s test revealed that myopes had significantly poorer fixation stability compared to emmetropes. Other pairwise comparisons were not significant. Next, we examined whether fixation stability was influenced by a deficit in a single meridian or both, by analyzing horizontal and vertical fixation dispersion. Horizontal and vertical fixation dispersion were also significantly greater in myopes: Horizontal dispersion: Kruskal-Wallis ANOVA on ranks H[2] = 39.8, p < 0.001; Vertical dispersion: Kruskal-Wallis ANOVA on ranks H[2] = 95.87, p < 0.001. Vergence stability, also varied significantly across groups Kruskal-Wallis ANOVA on ranks (H[2] = 8.77, p = 0.012). Dunn’s post hoc test indicated that myopes exhibited significantly greater vergence instability than emmetropes. No statistically significant differences were observed between hyperopic and emmetropic groups, nor between myopic and hyperopic groups.

**Table 1:** Fixation and vergence stability matrix showing median and interquartile range in myopes, hyperopes, and emmetropes.

Fixation and vergence stability matrix		
Fixation stability		
Groups	Median	IQR
Myopes	0.48*	0.71
Hyperopes	0.43	0.50
Emmetropes	0.42	0.46
Horizontal dispersion		
Myopes	0.48*	1.80
Hyperopes	0.332	0.39
Emmetropes	0.339	0.21
Vertical dispersion		
Myopes	0.43*	0.91
Hyperopes	0.28	0.19
Emmetropes	0.29	0.11
Vergence stability		
Myopes	0.57*	0.59
Hyperopes	0.48	0.76
Emmetropes	0.46	0.61

*p < 0.005 after Bonferroni correction.

IQR (Interquartile range).

### Fixational saccades

**Fig 6:**
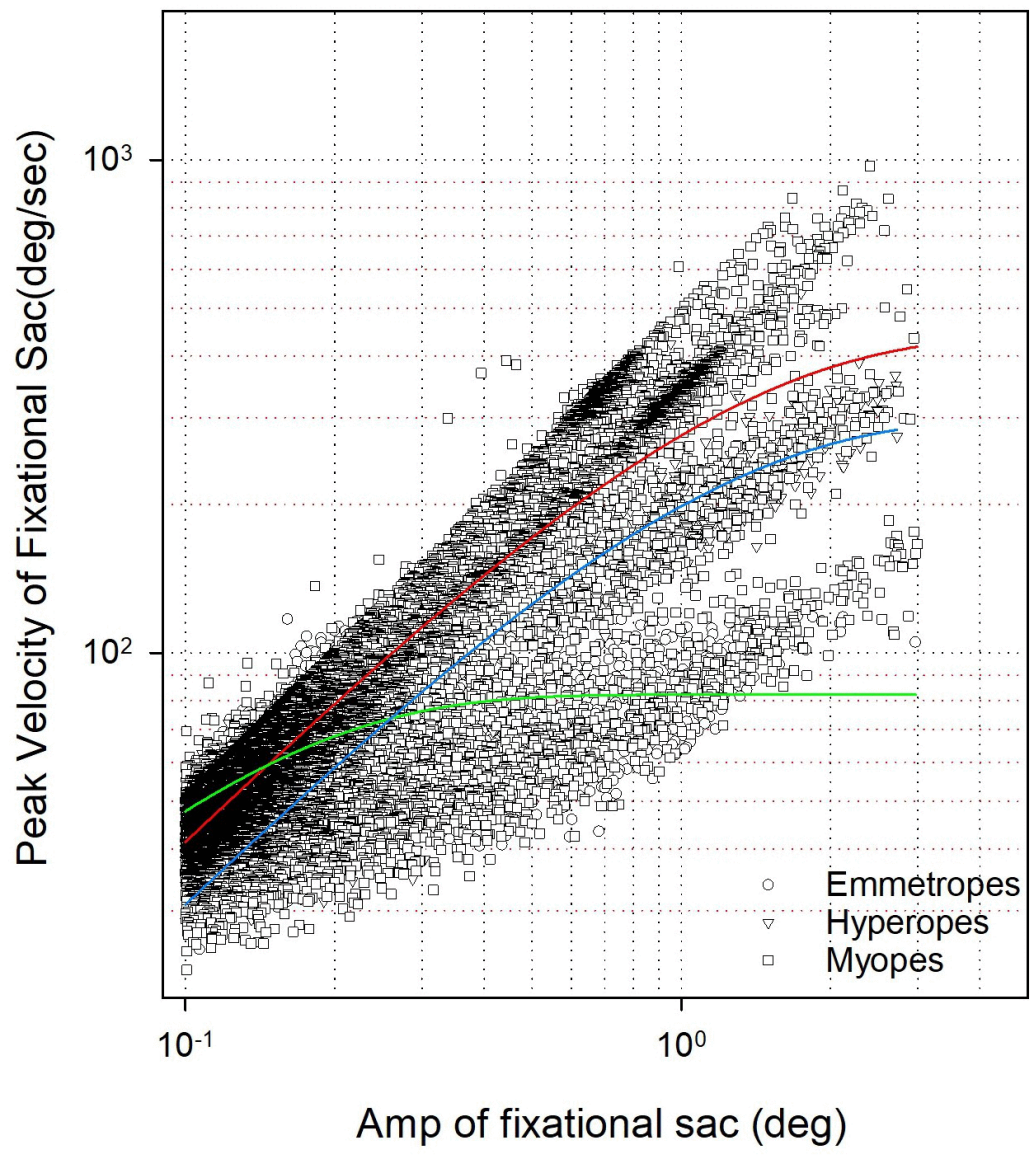
Scatter plot showing the relationship between amplitude (°) and peak velocity (°/s) of fixational saccades for myopes, hyperopes, and emmetropes. An exponential rise-to-maximum function was fitted to the data for each group. The fitted slopes are: Myopes (0.22), Hyperopes (0.14), and Emmetropes (0.38). The red line represents the myopic group, the blue line represents the hyperopic group, and the green line represents the emmetropic group.

**Fig 7:**
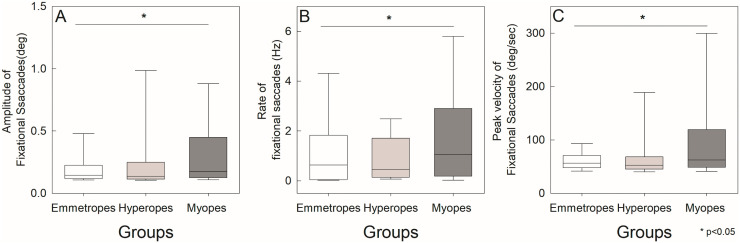
Box plots showing characteristics of fixational saccades in myopes, hyperopes, and emmetropes: (A) amplitude (°), (B) peak velocity (°/s), and (C) rate (Hz). The edges of the box represent the 25th and 75th percentiles; the median is shown by a solid black line. Whiskers extend to the most extreme non-outlier values. Asterisks (*) indicate statistically significant differences.

Now that we have examined fixation and vergence stability, we turned our attention to whether similar differences were present in fixational saccades. We began by analyzing the relationship between amplitude and peak velocity of fixational saccades occurring during steady fixation. The main sequence of fixational saccades (Fig 6) was consistent across all refractive groups, mirroring the characteristics typically observed in visually guided saccades. Given this consistency, we proceeded to compare the amplitude of fixational saccades across the different refractive groups to identify any group-specific variations. Myopes had a median amplitude of 0.18°(0.32°), significantly greater than hyperopes 0.14°(0.13°)and emmetropes 0.15°(0.11°). Amplitude of fixational saccades (Fig 7A) also showed significant group differences Kruskal-Wallis ANOVA on ranks (H[2] = 507.41, p < 0.001). Dunn’s post hoc test revealed myopes had significantly higher amplitude of fixational saccades than emmetropes and hyperopes. No significant difference was observed between hyperopes and emmetropes. Fixational saccade rate in myopes was 1.06(2.72) Hz, hyperopes had 0.45(1.57) Hz, and emmetropes had 0.64(1.78) Hz (Fig 7B), which differed significantly among groups, Kruskal-Wallis ANOVA on ranks (H[2] = 9.03, p = 0.011). Myopes exhibited a higher rate of fixational saccades than emmetropes, while no significant difference was observed between hyperopes and emmetropes. Peak velocity of fixational saccades in myopes were 62.41(70.86)°/sec, hyperopes had 52.38(22.96)°/sec, and emmetropes had 56.61(23.14)°/sec (Fig 7C). Peak velocity of fixational saccades also differed significantly, Kruskal-Wallis ANOVA on ranks (H[2] = 595.74, p < 0.001), with myopes showing the highest median values. Dunn’s test confirmed that myopes had significantly higher peak velocities than both hyperopes and emmetropes.

### Correlation analysis

**Fig 8:**
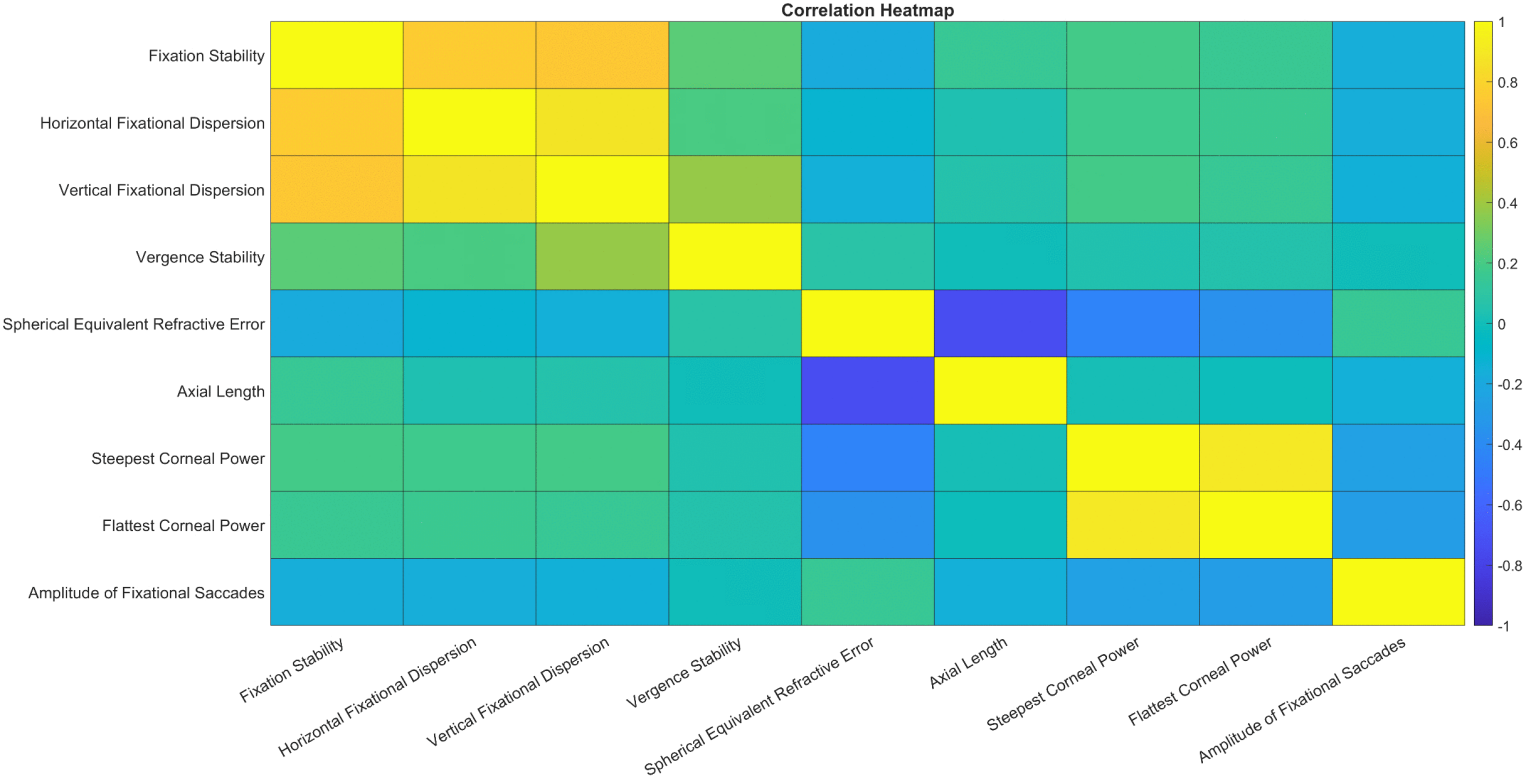
Heatmap showing correlations between fixation stability and various clinical and oculomotor parameters, including horizontal and vertical fixational dispersion, vergence stability, spherical equivalent refractive error, axial length, steep and flat corneal power, and amplitude of fixational saccades. Including correlation coefficient and * p < 0.05.

With all clinical refraction and oculomotor data available, we investigated potential clinical relevance by analyzing how fixation stability—quantified by BCEA—and its components relate to various clinical and oculomotor parameters. Specifically, we examined correlations between BCEA and horizontal and vertical fixational dispersion, vergence stability, spherical equivalent refractive error, axial length, steepest and flattest corneal power, and the amplitude of fixational saccades. Fig 8 presents a heatmap summarizing these relationships, offering a visual overview of how fixation stability interacts with both clinical and oculomotor variables. There was no strong correlation between any clinical measurements and BCEA.

## Discussion

This study investigated oculomotor behavior across three refractive groups—myopes, hyperopes, and emmetropes—with a particular focus on visually guided saccades, fixation stability, vergence stability, and fixational eye movements. While visually guided saccadic metrics such as latency, gain, and peak velocity did not differ significantly among the groups, several key distinctions emerged in fixational behavior, particularly among myopic participants.

Fixation stability and dispersion varied significantly across refractive groups. Myopes exhibited greater horizontal and vertical dispersion of fixational eye movements, suggesting reduced coordination or more variable control during fixation. The larger BCEA observed in myopes further supports the notion of diminished fixation precision. As we know increased axial length in myopic eyes can alter retinal image quality and binocular coordination, affecting oculomotor control and fixation stability. High myopia is associated with posterior segment deformation, which may impair central fixation and lead to larger fixational eye movements such as microsaccades and drifts [54]. These changes disrupt spatiotemporal modulation of retinal input, reducing sensitivity to high spatial frequencies and altering visual processing [55]. Fixation stability declines with increasing myopia severity, particularly under blur or low luminance, indicating contributions from both optical and neural factors [36,56]. Collectively, fixation instability in myopes likely results from biomechanical alterations, degraded visual feedback, and adaptive changes in oculomotor control. Findings of our study are consistent with previous research indicating that myopia is associated with less stable fixation, even when corrected [36,32]. Coletta et al. reported higher BCEA values in corrected myopic than hyperopic groups using a 0.5° black cross target [34]. According to Pirdankar and Das and Thaler et al, both the shape and size of the fixation target influence fixation stability [57,58]. While our target was the same size as in Coletta’s study 0.5°, the shape differed there was a black cross whereas our study employed a 0.5° disk-shaped target [34]. Regardless of the target shape, myopes exhibited greater fixation instability than hyperopes. We did not find a significant correlation between axial length and BCEA—similar to Coletta et al.’s findings—our data did reveal greater horizontal and vertical dispersion in myopes, reinforcing the trend they observed [34]. Zheleznyak et al. reported that myopes tend to perceive vertically elongated blur, while hyperopes and emmetropes perceive horizontally elongated blur [59]. Whether perceptual differences in blur orientation influence oculomotor control remains an open and intriguing question. In our study, myopes exhibited greater fixation dispersion in both axes, with horizontal dispersion exceeding vertical. Overall, fixation stability was significantly correlated with both horizontal and vertical fixational dispersion. When examining subgroups based on the magnitude of spherical equivalent refractive error, only the low myopic and hyperopic groups showed a significant correlation with fixation stability. Future studies should focus on comparing high myopes and high hyperopes in greater detail, with a larger sample size.

Vergence BCEA also differed across the three groups, suggesting that fine oculomotor control during fixation is more susceptible to refractive influences, particularly in myopia. Although this differs from what Ukwade and Bedell reported, the variability of vergence with induced optical blur was not significant [60]. From a theoretical standpoint, these findings support the hypothesis that refractive error—especially myopia—is associated with specific alterations in fine oculomotor control. Practically, these differences may have implications for tasks requiring sustained visual attention and precision, such as reading, digital screen use, and fine motor coordination. Given projections that over half of the global population will be myopic by 2050, understanding the oculomotor consequences of refractive error is increasingly important for clinical assessment, visual ergonomics, and intervention strategies [61].

Fixational saccade dynamics followed the expected pattern, with peak velocity increasing as saccade amplitude increased, representing the positive curvilinear relationship between these parameters. While this trend was consistent across all refractive groups, we observed an earlier saturation of the main sequence slope in emmetropes compared to myopes and hyperopes. This pattern may indicate more efficient oculomotor control and favorable biomechanical properties in the normal eye. In contrast, structural changes associated with refractive errors, such as increased axial length in myopia or altered ocular geometry in hyperopia, could affect muscle force transmission and neural programming, leading to subtle differences in saccade dynamics [62–65]. Our findings also showed myopes exhibited significantly greater amplitude, higher rates, and elevated peak velocities of fixational saccades compared to emmetropes. These results suggest increased oculomotor instability or compensatory microsaccadic activity in myopia. These findings are consistent with previous studies by Coletta et al., Ghasia and Shaikh, and others, which reported increased fixation instability and microsaccadic amplitudes in myopic individuals [32,34,36]. Such alterations may be attributed to axial elongation in myopia, which could disrupt proprioceptive feedback or cortical mechanisms involved in fine oculomotor control. Interestingly, while Ghasia et al. found a significant correlation between increased fixational saccade amplitude and spherical equivalent refractive error after correction, we did not see similar relationship. Based on our findings, we propose that larger BCEA values in ametropes, particularly myopes, may reflect increased neural noise rather than differences in basic clinical measurements.

Despite these insights, several limitations should be acknowledged. First, the relatively small sample size of the hyperopic group limits the statistical power to detect subtle differences. Second, the cross-sectional design precludes causal inferences or insights into the developmental trajectories of oculomotor behavior. Third, all participants in our study wore soft contact lenses to ensure optimal refractive correction. We did not control for the specific types of lenses worn. There may be concerns regarding potential contact lens slippage and its impact on eye tracking measurements. While contact lenses are not fixed and may shift slightly on the eye surface, our video-based eye tracker uses both corneal light reflex and pupil center detection algorithms. Minor lens movement could theoretically affect amplitude measurements via corneal reflection, but in our case, this influence was minimal due to the dual-tracking approach. Moreover, this effect was consistent across all refractive groups and cannot be attributed to any single group.

Future research should explore whether the observed oculomotor differences are adaptive responses or maladaptive consequences of refractive error. Longitudinal studies could help determine whether these traits precede or follow the onset and progression of myopia. Additionally, the role of blur adaptation in shaping fixation behavior warrants further investigation. Whether perceptual differences in blur orientation influence oculomotor control remains an important and unanswered question. Finally, hyperopes remain underrepresented in oculomotor research despite their clinical relevance. Their reliance on accommodative compensation may mask underlying oculomotor deficits. Future studies should include larger hyperopic samples, including a younger population, and consider cycloplegic refraction to investigate accommodative-vergence interactions.

## Conclusion

This study highlights distinct differences in oculomotor behavior among individuals with varying refractive errors, particularly in fixational eye movements. While visually guided saccadic metrics remained consistent across myopes, hyperopes, and emmetropes, myopes exhibited significantly greater fixation instability, vergence instability, including increased fixational saccades amplitude, fixational saccades rate, and dispersion of fixational eye movements. These findings suggest that myopia may be associated with altered fine oculomotor control, potentially impacting tasks that require sustained visual attention and precision. Understanding these differences is essential for advancing clinical diagnostics, tailoring visual training programs, and informing public health strategies in the context of the global rise in refractive errors.
